# Functional Bionanocomposite Fibers of Chitosan Filled with Cellulose Nanofibers Obtained by Gel Spinning

**DOI:** 10.3390/polym13101563

**Published:** 2021-05-13

**Authors:** Sofia Marquez-Bravo, Ingo Doench, Pamela Molina, Flor Estefany Bentley, Arnaud Kamdem Tamo, Renaud Passieux, Francisco Lossada, Laurent David, Anayancy Osorio-Madrazo

**Affiliations:** 1 Institute of Microsystems Engineering IMTEK, University of Freiburg, 79110 Freiburg, Germany; sofia.marquez@imtek.uni-freiburg.de (S.M.-B.); ingo.doench@imtek.uni-freiburg.de (I.D.); molina@tf.uni-freiburg.de (P.M.); estefany.bentley@imtek.uni-freiburg.de (F.E.B.); arnaud.kamdem@imtek.uni-freiburg.de (A.K.T.); 2 Freiburg Materials Research Center FMF, University of Freiburg, 79104 Freiburg, Germany; 3 Freiburg Center for Interactive Materials and Bioinspired Technologies FIT, University of Freiburg, 79110 Freiburg, Germany; 4 Laboratoire Ingénierie des Matériaux Polymères IMP, CNRS UMR 5223, University of Lyon, University Claude Bernard Lyon 1, CEDEX, 69622 Villeurbanne, France; renaud.passieux@etu.univ-lyon1.fr (R.P.); laurent.david@univ-lyon1.fr (L.D.); 5 Department of Chemistry, University of Mainz, 55128 Mainz, Germany; francisco.lossada@uni-mainz.de

**Keywords:** polymer fiber yarns, bio-nanocomposites, chitosan, cellulose nanofibers, gel/wet spinning, mechanical properties

## Abstract

Extremely high mechanical performance spun bionanocomposite fibers of chitosan (CHI), and cellulose nanofibers (CNFs) were successfully achieved by gel spinning of CHI aqueous viscous formulations filled with CNFs. The microstructural characterization of the fibers by X-ray diffraction revealed the crystallization of the CHI polymer chains into anhydrous chitosan allomorph. The spinning process combining acidic–basic–neutralization–stretching–drying steps allowed obtaining CHI/CNF composite fibers of high crystallinity, with enhanced effect at incorporating the CNFs. Chitosan crystallization seems to be promoted by the presence of cellulose nanofibers, serving as nucleation sites for the growing of CHI crystals. Moreover, the preferential orientation of both CNFs and CHI crystals along the spun fiber direction was revealed in the two-dimensional X-ray diffraction patterns. By increasing the CNF amount up to the optimum concentration of 0.4 wt % in the viscous CHI/CNF collodion, Young’s modulus of the spun fibers significantly increased up to 8 GPa. Similarly, the stress at break and the yield stress drastically increased from 115 to 163 MPa, and from 67 to 119 MPa, respectively, by adding only 0.4 wt % of CNFs into a collodion solution containing 4 wt % of chitosan. The toughness of the CHI-based fibers thereby increased from 5 to 9 MJ.m^−3^. For higher CNFs contents like 0.5 wt %, the high mechanical performance of the CHI/CNF composite fibers was still observed, but with a slight worsening of the mechanical parameters, which may be related to a minor disruption of the CHI matrix hydrogel network constituting the collodion and gel fiber, as precursor state for the dry fiber formation. Finally, the rheological behavior observed for the different CHI/CNF viscous collodions and the obtained structural, thermal and mechanical properties results revealed an optimum matrix/filler compatibility and interface when adding 0.4 wt % of nanofibrillated cellulose (CNF) into 4 wt % CHI formulations, yielding functional bionanocomposite fibers of outstanding mechanical properties.

## 1. Introduction

Fibers are structural components with a wide range of applications in medicine, biotechnology, textiles and industrial materials [1]. In biomedicine, the oldest and most common examples of fibers are surgical sutures and medical textile structures. However, fibers have also been used to develop many other products, such as fibrous scaffolds for tissue engineering [2,3,4,5], fibrous porous media [6,7], knitted fabrics, etc. The vast selection of materials, which are used for routine surgical sutures is mainly limited to synthetic polymers, such as polyethylene terephthalate (PET), polyglycolide (PGA), polyamide 6.6 (PA 6.6), polypropylene (PP), polyvinyl alcohol (PVA), para-aramid and ultra-high molecular weight polyethylene (UHMWPE). The fiber spinning process should allow forming fibers with high modulus and high strength. Fine control over the mechanical properties is gained via stretching ratio, stretching temperature and thermal treatments, impacting the crystalline microstructure of the polymers with high molecular weight [8]. However, synthetic polymers maintain strong limitations, such as reduced biocompatibility and resorbability, increasing the risks of inflammation and infections, particularly when the implants are used for long-term applications.

Recently, there has been an increased interest in using natural polymers (cellulose, chitin, chitosan, hyaluronic acid, alginates, etc.) for biomedical and bioengineering applications because of their biocompatibility resorbability, availability, versatility and possible similarity to the structure of the extracellular matrix [9]. Focusing on chitosan, this is a polysaccharide of D-glucosamine and N-acetylated-D-glucosamine units linked by β(1 → 4) glycosidic bonds, which is mainly produced from the deacetylation of chitin this latter mainly extracted from crustacean shells. High-grade chitosans can also be obtained from the deacetylation of β-chitin found in cephalopod endoskeletons [10]. Besides being obtained from one of the most abundant natural polysaccharides, chitosan offers excellent properties, such as bioactivity (e.g., wound healing), biocompatibility, biodegradability, low toxicity and hemostatic activity [11,12,13,14,15,16]. For this reason, huge efforts have been made to exploit the properties of chitosan fibers [1,17,18,19] and be able to adapt them into biomedical and bioengineering applications, such as scaffolds for tissue engineering, suture threads, drug delivery systems, wound dressings, separation membranes, antibacterial coatings [10,20,21,22]. A methodology used to obtain fibers from polymeric collodion solutions is through gel/wet spinning, for example, viscous chitosan solutions. However, such chitosan yarns often offer reduced mechanical performance in their dry state [23]. Such a feature is a great limitation for further processing the fibers in knitted textiles, hindering their potential applications [17].

Similarly, cellulose is a linear homopolymer polysaccharide of β(1 → 4)-D-glucose linked units with a practically inexhaustible source in Earth, especially in the plant biomass. Cellulose can be processed in nanometric fibril form (cellulose nanofibers) [24,25]. Two main classes of cellulose nanofibrils can be distinguished, namely the cellulose whisker nanocrystals (CNW) [26,27,28] and the nanofibrillated cellulose (CNF) [29,30]. Both types of nanocellulose offer low-density, low-cost, high mechanical properties, specific barrier properties, and low thermal expansion [31]. As a reinforcing agent, nanocellulose is of particular interest due to its nanometric size, high aspect ratio, high crystallinity, and ability to form H-bonds inducing interfibrillar network structures or intermolecular interactions, leading to outstanding mechanical properties when combined with polymeric matrices [14,15,27,30,32,33,34,35,36].

Chitosan has relatively low mechanical performance compared to synthetic polymers. Its mechanical behavior can be improved by adding synthetic polymers, plasticizers or mineral fillers. However, its biocompatibility can be affected by such formulations. For this reason, the main objective of this work is to obtain fully bio-based and biocompatible nanocomposite fibers with high mechanical performance, reinforcing a chitosan matrix with CNFs, through a gel spinning process. The resulting composite fibers should combine the excellent biological properties of chitosan with the outstanding mechanical properties of cellulose nanofibers, thus allowing them to be useful as a functional material for biomedical applications, such as knitted fabrics, suture threads, adhesives, wound dressings, and even tissue engineering scaffolds, among others. Recent works reported the interest of chitosan composites reinforced with nanocellulose [14,15,37], in which the incorporation of a minor amount of CNFs into a chitosan matrix can produce huge improvement in the functional, mechanical and barrier properties of chitosan biomaterials. Other authors also proposed to use chitin/chitosan-based matrices reinforced with cellulose [19,38,39,40,41,42,43]. Wu et al. [38] prepared fiber filaments of regenerated chitin (RC) reinforced with bacterial cellulose nanocrystals (BCNC), showing a strong increase of tensile resistance related to the presence of BCNC. A study of enzymatic degradation showed that BCNC/RC present good biodegradability without cytotoxicity and even promote cellular proliferation. In vivo tests on a mouse model demonstrated that BCNC/RC showed good biocompatibility combined with mechanical performance and promoted wound healing, offering the potential to be used in surgical sutures. Zhu et al. [44] obtained fibers of chitosan and cellulose (cotton linter pulp) with a common solvent, i.e., 4.5 wt % in LiOH, 7.5 wt % in KOH and 11.5 wt % in aqueous urea. The fibers exhibited good mechanical properties due to the good miscibility and combination of their components through hydrogen bond interactions. Additionally, they exhibited memory shape behavior under water and acid stimulation. Nevertheless, biomedical applications may be difficult due to toxicity of Li^+^ ions. Definitively, for an application in biomedical engineering, toxic solvents need to be limited. Interestingly, Cai et al. [45] presented fibers of nanocellulose and chitosan by inducing interfacial polyelectrolyte complexation of chitosan with TEMPO-oxidized cellulose nanofibrils and further synergistic complexation with Ca^2+^ ions. Improvements in the strength and toughness of the composites would be due to the interfacial interactions between CNF nanofibers and CHI molecules. Combined with the excellent mechanical properties, they obtained good biocompatibility and concluded with the potential possibility to use them as surgical sutures, biosensors and structural reinforcing agents. However, the interfacial association process still needs to be upscaled into a true industrial process [39]. Peniche et al. [19] produced chitosan-based fibers with proteins (bovine serum albumin BSA) via the wet-spinning process. The mentioned fibers showed suitable mechanical properties to be explored as reinforcement within hydrogels. Doench et al. [14,15] prepared chitosan hydrogels reinforced with CNF for the regeneration and repair of mechanically demanding tissues like the *annulus fibrosus* of the intervertebral disc. They concluded that the addition of CNF significantly improves the mechanical properties of the composite hydrogels. Moreover, the in situ gelation of CHI/CNF precursor suspension could be used for applications in intervertebral disc nucleosupplementation [15]. Additionally, they carried out ex vivo experiments in porcine models, evidencing that the implantation of these composite hydrogels within fenestrated (defective) discs helps to restore their biomechanics [14,46]. Solid forms resulting from the drying of these composite hydrogels should also be of interest for their combined mechanical properties and biocompatibility. Finally, Azevedo et al. [47] made small hollow tubes with mixtures of methylol cellulose and chitosan as low diameter vascular substitutes, reacting cellulose with paraformaldehyde in DMSO, and mixing with a chitosan hydrochloride aqueous solution. These tubes exhibited good mechanical properties and cell compatibility, highlighting the need of further investigations as a biocompatible synthetic candidate for coronary artery bypass graft applications, even if again, an organic solvent-free, toxic compound-free is to be preferred for biomedical applications. Nevertheless, both chitosan and cellulose offer complementary physico-chemical structure, biocompatibility, renewability. Thus, in the quest of associating these polysaccharides through a process in aqueous media without chemical modification and toxic reactants or complexants, a gel spinning process is explored for the development of nanocomposite fibers, consisting of CHI and nanofibrillated cellulose (CNFs). In particular, by varying the concentration of both constituents, we evaluate the properties of the CHI/CNF hydrogel precursor suspension and final dry fibers, for the achievement of functional bionanocomposite fibers which could find potential application in the engineering of fiber-containing mechanically demanding tissues like the annulus fibrosus region of the intervertebral disc, among other tissues.

## 2. Materials and Methods

### 2.1. Chitosan (CHI)

Chitosan (Type: CHITOSAN 144, Batch No. 20120926) from squid pen chitin was supplied by Mahtani Chitosan (Veraval, Gujarat, India). The CHI degree of acetylation (DA) was 2.5%. It was determined by H^1^ NMR spectroscopy following the methodology of Hirai et al. [48]. The chitosan (10 mg) was dissolved in 1 mL of D_2_O acidified with 5 μL of concentrated HCl (12 M). The measurement was performed on a Bruker ALS 300 spectrometer (Bruker GmbH, Ettlingen, Germany) (300 MHz) at 298 K. The CHI molecular weight was determined by size exclusion chromatography (SEC) coupled to multi-angle laser light scattering (MALLS) as previously described [49]. The number (Mn) and weight–average molecular weight (Mw) of CHI were 4.1 × 10^5^ g/mol (±6.4%) and 6.1 × 10^5^ g/mol (±9.6%), respectively, with a polydispersity index Ip = 1.49 (±11.6%).

### 2.2. Cellulose Nanofibers (CNF)

Gel-like suspensions of nanofibrillated cellulose (CNF) were obtained from bleached pine sulfite dissolving pulp at the Centre Technique du Papier (CTP, Grenoble, France) by a mechano-enzymatic method adapted from Pääkkö et al. (2007) [50]. Before 1 h incubation at 50 °C with a solution of endoglucanase FiberCare R^®^ (Novozymes Biologicals, Paris, France) at pH 5.0, the pulp was refined at 4.5% consistency with a 12” single disk refiner for 25 min. The digested samples were further refined to obtain a pulp suspension of SR (Schopper-Riegler) number higher than 80 and mean fiber length smaller than 300 µm. A quantity of 2 wt % fiber suspensions was processed with an Ariete homogenizer, involving one pass at 1000 bar followed by 3 passes at 1500 bars. The obtained CNFs displayed a surface charge density of 40–80 mmol/kg. In other words, they were weakly charged with carboxylate moieties. The morphology of the cellulose nanofibers was characterized by atomic force microscopy (AFM) as reported in our previous works [51], which revealed an entangled network of interconnected nanofibrils with an average width of 35.2 ± 8.1 nm and bundles up to 100 nm width.

### 2.3. Preparation of CNF-Filled Chitosan Suspensions, and CHI Solution

Viscous collodions of chitosan dissolved in a weakly amount of acetic acid, then filled with cellulose nanofibers as above, were prepared to be used to produce CNF/CHI bionanocomposite yarns by gel spinning. A fine powder of chitosan (CHI) at 4 wt % was mixed with CNFs in water at a given CNF content (0.2; 0.3; 0.4 or 0.5 wt %). The dispersions were sonicated with a SONOPULS ultrasonic homogenizer (Bandelin electronic GmbH, Berlin, Germany) for 5 min at 40% amplitude. Then, acetic acid was added in stoichiometric amounts to protonate the amine moieties of chitosan (DA = 2.5%) to solubilize the chitosan. The mixture was kept under mechanical stirring overnight. Finally, CHI/CNF aqueous suspensions and reference CHI viscous solutions were obtained as collodion for further fiber spinning.

### 2.4. Shear Rheological Tests on CHI/CNF Formulations

A MARS II Rheometer from Thermo Fisher Scientific fitted with a cone-plate flow geometry and titanium plate (PP35 Ti) was used to characterize the rheological behavior of the different chitosan/cellulose nanofibers CHI/CNF viscous formulations at 25 °C, with a gap size of 1 mm and a solvent trap to prevent its drying or evaporation. The cone-plate geometry (diameter: 35 mm; angle: 4°) allows ensuring a uniform shearing to the sample. The analysis was performed in triplicate in continuous mode in a shear rate range from 0.005 to 1000 s^−1^. The flow diagrams, namely the plots of the steady-state shear viscosity vs. shear rate, of the CHI/CNF collodion formulations were obtained.

### 2.5. Gel Spinning of CHI/CNF Viscous Formulations into CHI/CNF Bionanocomposite Fibers

**Figure 1:**
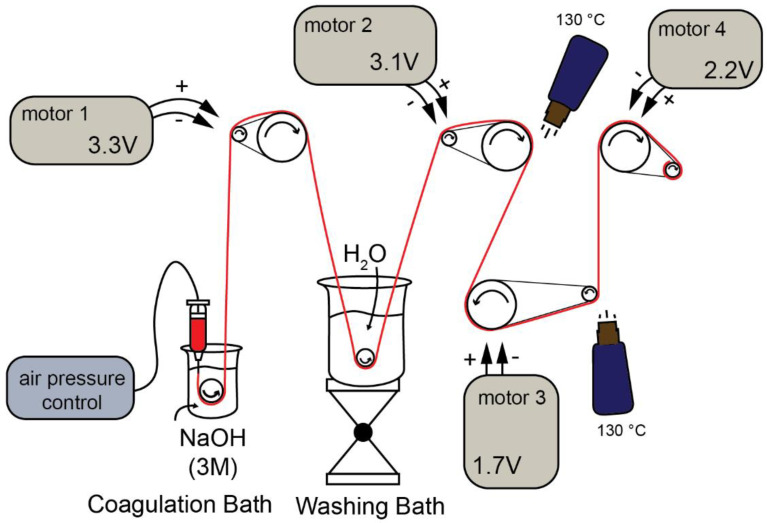
Schematic illustration of the wet/gel spinning setup to achieve nanocomposite fiber yarns of chitosan (CHI) filled with cellulose nanofibers (CNF).

In Figure 1, the setup to obtain CHI/CNF fiber yarns by gel spinning is schematized. It consisted of a syringe barrel of 30 mL, which contained the CNF-filled CHI viscous suspension (or CHI viscous solution), connected to a controlled air-pressure clip of a Performus I Nordson EFD dispenser connected to compressed air. The syringe was connected to a conic needle with a tip diameter of 0.58 mm (gauge 20, pink, Nordson EDF). The CHI/CNF viscous extrudate enters the coagulation bath (3M NaOH), which surface was located at a distance of ~2 mm from the needle tip. A ceramic bobbin roller (V-shape pink ceramic diabolo, Petit Spare Parts, Aubenas Cedex, France) was immersed in the coagulation bath, on which the coagulated hydrogel macrofilament is pulled out with the aid of a DC motor, which rotation speed (Table 1) is controlled by the input voltage fixed by the power supply (BaseTech BT-153, Hamburg. Bermany) (Figure 1). Afterward, the hydrogel macrofiber passed through a washing bath of deionized water, where another ceramic roller was immersed, from which the macrofiber was stretched and pulled out by the spinning motor 2. A spinning motor 3 and a spinning motor 4 allowed the further stretching of the fibers, their drying and finally their collection on a bobbin. The summary of the linear bobbin speeds supported by the different motors is related in Table 1. The drying of the fiber is a critical step and was supported by heat guns placed in the setup near the spinning motors 3 and 4, allowing heating at 130 °C. The spinning setup runs for 10 min, which yields fibers of around 25 m in length. After preliminary works dealing with the preparation of viscous CHI solutions and CHI/CNF suspensions of varied CHI and CNF compositions, the appropriate range of concentrations of the constituents was fixed to achieve the stable spinning of CHI/CNF systems. Composite fibers of CHI/CNF were processed through gel spinning of viscous collodions of a given CHI concentration of 4 wt %, with varied CNF contents of 0.2, 0.3, 0.4 and 0.5 wt %.

### 2.6. Characterization of the CHI/CNF Bionanocomposite Spun Fibers

#### 2.6.1. Scanning Electron Microscopy (SEM)

The fracture and the lateral surfaces of the CHI/CNF spun fibers were observed using a FEI Scios DualBeam FIB/SEM microscope at an accelerated voltage of 5 kV after sputter-coating an ultrathin Au layer.

#### 2.6.2. Fourier Transform Infrared (FTIR)

The CHI/CNF spun fibers were characterized by FTIR in attenuated total reflectance (ATR) mode using an FTIR-ATR Spectrum 65 spectrometer (PerkinElmer, Besweiler, Germany). Spectra in the range of 400–4000 cm^−1^ were recorded by the accumulation of 64 scans. Reference spectra of the starting chitosan powder and the nanofibrillated cellulose (CNF) were also recorded.

#### 2.6.3. Wide Angle X-ray Scattering (WAXS)

**Figure 2:**
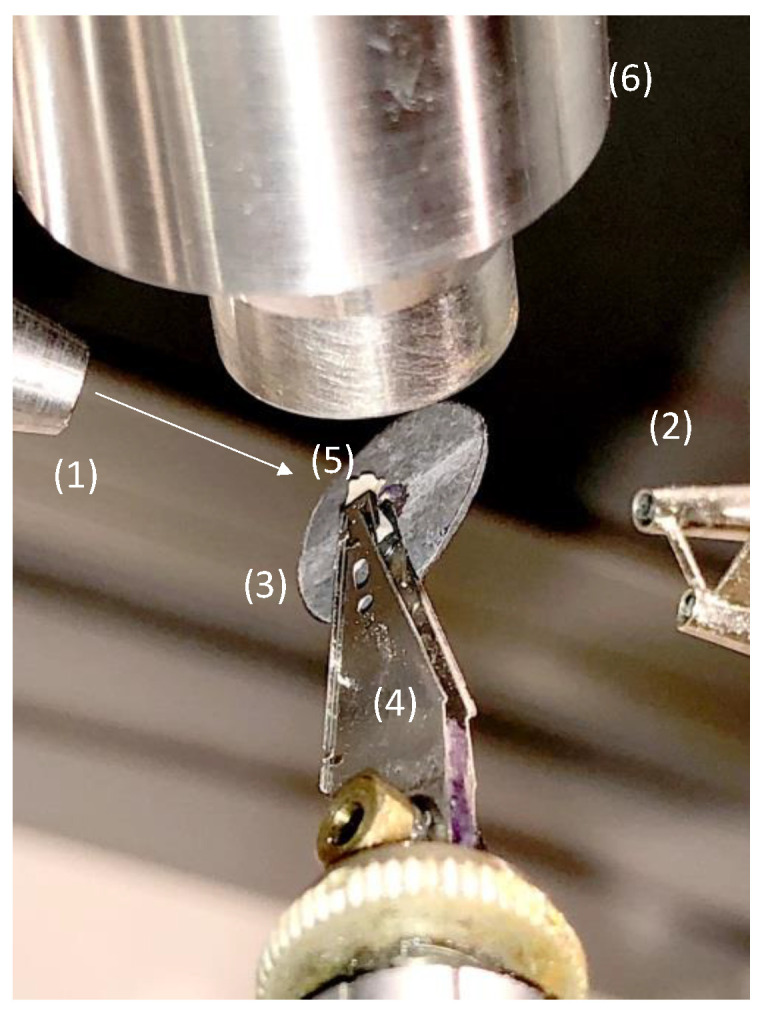
Photograph of the fiber sample holder used for X-ray scattering analysis in transmission mode in the Gemini A Ultra diffractometer. (1) X-ray delivery tube (CuKα), (2) two-well beamstop, (3) lead washer shield, (4) elastic pliers (collected from a 3.25″ hard disk), (5) sample, (6) cryostat nozzle (courtesy of Ruben Vera, *Centre de Diffraction Henri Longchambon*, University Claude Bernard Lyon 1, University of Lyon, France).

For the characterization of the CHI/CNF spun fibers by wide-angle X-ray scattering (WAXS), the samples were entrapped on a home-made lead sample holder with a hollow center, as shown in Figure 2, to allow the X-ray beam to pass through a bundle of 3 aligned strains cut from the same spun fiber. Two-dimensional WAXS patterns of the CHI/CNF fibers were recorded in transmission mode at a scattering vector *q* range between 0.5 to 3.5 Å^−1^ in a Gemini A Ultra diffractometer (Agilent Technologies XRD Products, Oxfordshire, UK), with an Atlas CCD detector and using the copper K-alpha radiation. The data collection consisted of 5 images, each with 17 min exposure time. All 5 images were averaged for each sample, and the azimuthal *q*-scans were calculated after averaging around the incidence center. Transmission coefficients were estimated for background subtraction.

#### 2.6.4. Thermogravimetric Analysis (TGA)

Thermogravimetric analysis of the CHI/CNF spun fibers was performed on a STA449 F5 Netzsch thermal gravimetric analyzer. Approximatively 10 mg of cut fibers were weighed in a platinum pan and heated from room temperature (~25 °C) up to 650 °C at a heating rate of 10 °C/min, under nitrogen atmosphere with a flow rate of 100 mL/min.

#### 2.6.5. Dynamic Mechanical Thermal Analysis (DMTA)

Dynamic mechanical thermal analysis measurements of the CHI/CNF spun fibers were performed using an MCR702 multidrive (Anton Paar GmbH, Ostfildern, Germany) with tensile loading. The experiments were performed with 0.05 N of preload and 0.03 N of test force at a frequency of 1 Hz. The tested fibers had a length of 8 mm, and the scanned temperature ranged from 25 to 250 °C at a heating rate of 3 °C/min in air. Three samples were measured for each condition.

#### 2.6.6. Microtensile Testing

Microtensile tests of the CHI/CNF spun fibers were performed using a DEBEN minitester equipped with a 20 N load cell. All tests were carried out at room temperature and a constant strain rate of 0.5 mm/min. Fiber segments with 8 mm length were cut and fixed to the tester with a length between clamps set to 3 mm, corresponding to the initial fiber tensed length (*L_0_*). The nominal stress *σ* was calculated as the ratio of the applied force *F* to the initial cross-sectional area *A* of the fiber (*σ* = *F*/*A*), whose dimension was determined using a light optical microscope (Olympus BX Series, Hamburg, Germany). The nominal strain ε was expressed as the ratio of the extension of the fiber with respect to its initial length *L_0_* (ε = Δ*L*/*L_0_* = (*L* − *L_0_*)/ *L_0_*). The Young’s modulus (*E*), yield stress (*σ_y_*) and strain (*ε_y_*), ultimate stress (*σ_b_*) and strain at break (*ε_b_*) were determined from the obtained stress–strain curves, considering at least ten replicates (*n* = 10) in the measurement, for each CHI/CNF spun fiber formulation.

## 3. Results

### 3.1. Rheological Behavior of CNF-Filled CHI Suspensions

**Figure 3:**
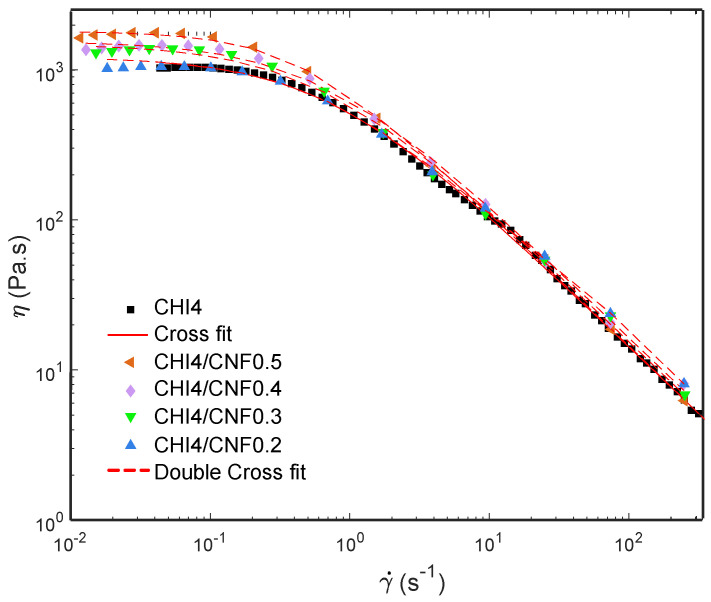
Flow diagrams showing the evolution of the viscosity (*η*) vs. shear rate ($\dot{\gamma }$) of CNF-filled CHI suspensions and CHI reference solution, also displaying the modeling of the rheology data.

Figure 3 shows the flow diagrams, i.e., viscosity (*η*) vs. the shear rate ($\dot{\gamma }$) of CHI/CNF suspensions at a chitosan concentration of 4 wt %, for different added CNF contents (0.2, 0.3, 0.4 and 0.5 wt %). All systems display a plateau value at low shear rates. Such Newtonian viscosity of the CHI/CNF suspensions increases with the CNF content. This is explained by the formation of a transient network of CNFs, possibly bridged by chitosan chains. This behavior should indicate a good interaction between the CNFs and the CHI macromolecules [15,17,52]. At higher shear rates, the typical non-Newtonian shear-thinning behavior of the CHI solution is observed, with a low-power decrease of viscosity with increasing shear rates [53]. The flow behavior is related to the disentanglement of the chitosan chains, also inducing chain orientation. Overall, flow-induced orientation tends to form an anisotropic structure under the action of a shear field [17,54]. In the highest shear range, the viscosity similarly decreased in the pure CHI solution and in the CNF/CHI suspensions, revealing that the shear-thinning behavior in the latter is governed by the disentanglement of CHI chains and practically not affected by the presence of the CNFs, which should get oriented in the flow direction [53,55].

A three-parameter Cross law (Equation (1)) was used to model the flow diagrams (*η* vs. $\dot{\gamma }=\frac{d\gamma }{dt})$ of the naked chitosan viscous solution [15,56,57,58]:

(1) $$\frac{\eta_{0,CHI}}{1+{\left(\dot{\gamma }\tau_{CHI}\right)}^{p_{CHI}}}$$

where *η*_0_ is the Newtonian or zero-shear viscosity, *p* is equal to *1-n* with *n* being the flow behavior index, and *τ* is the relaxation time for polymer chain disentanglements. Similar rheological behavior was observed for the suspension containing the lowest CNF content of 0.2 wt %. Then, the CNF-filled CHI viscous suspensions with increased CNF concentration exhibited more complex flow diagrams, with higher Newtonian viscosities measured in the low shear rate range ($\dot{\gamma }<0.1\;s^{-1}$), which was more evident for the highest CNF contents and a shear-thinning occurring in the higher shear rate regime (Figure 3). Thus, a double Cross law (Equation (2)) could model the two-step flow diagrams of the CHI/CNF suspensions with CNF contents beyond 0.2 wt % [15]:

(2) $$\frac{\eta_{0,CHI}}{1+{\left(\dot{\gamma }\tau_{CHI}\right)}^{p_{CHI}}}+\frac{\eta_{0,CNF}}{1+{\left(\dot{\gamma }\tau_{CNF}\right)}^{p_{CNF}}}$$

where *η_0,CNF_*, *τ_CNF_*, and *p*_CNF_ = 1 − *n_CNF_* are the flow parameters associated with the disruption of the CNF network at low shear rates, possibly due to un-bridging CNFs in the CHI/CNF system [15]. The model fit of the rheological results is also shown in Figure 3. The fitting used a Levenberg–Marquardt nonlinear regression algorithm in the Octave 4.4.0 programming environment. Table 2 shows the flow parameters obtained from the double Cross fitting for the CHI/CNF suspensions, as well as the Newtonian viscosity *η_0,CNF_*, the relaxation time *τ_CNF_*, and the exponent *p*_CNF_ = 1 − *n_CNF_*. To summarize, in the CHI/CNF formulations, two different relaxation phenomena should be at work. In the pure CHI reference solutions, the main chain relaxation, at high shear rates, corresponds to the disentanglement of the CHI chains, with a relaxation time of around 1 s in a viscous solution of 4 wt % CHI. When adding the CNFs, a second relaxation occurs with slightly longer relaxation times of the order of 2–3 s (Table 2). In addition, the amplitude of the low shear rate relaxation mode increases with the CNF content, and the longer relaxation time could be related to motions of larger moieties and interacting chitosan chains with slower dynamics. Electrostatic interactions could establish between the positively charged chitosan (polycation) and the weakly negatively charged CNF (polyanion), as these latter have a surface charge density of 40–80 mmol/kg due to a low amount of carboxylate moieties present in the nanofibrils surface [59,60]. Other interactions probably play a role in the interaction between cellulose and chitosan, namely H-bonds and hydrophobic interactions. Thus, CHI polymer chains may absorb on the CNF surface and contribute to bridging the nanofibers [61,62], resulting in entanglement formation between the adsorbed chains and the other neighbor CHI chains in the solution. As the nanofibers present a very large surface, [63] the addition of CNF even at low concentrations is likely to impact the dynamics of the chitosan chains and result in forming a weak CNF network.

### 3.2. Processing of CHI/CNF Collodion into Fiber Yarns and Fiber Morphology

**Figure 4:**
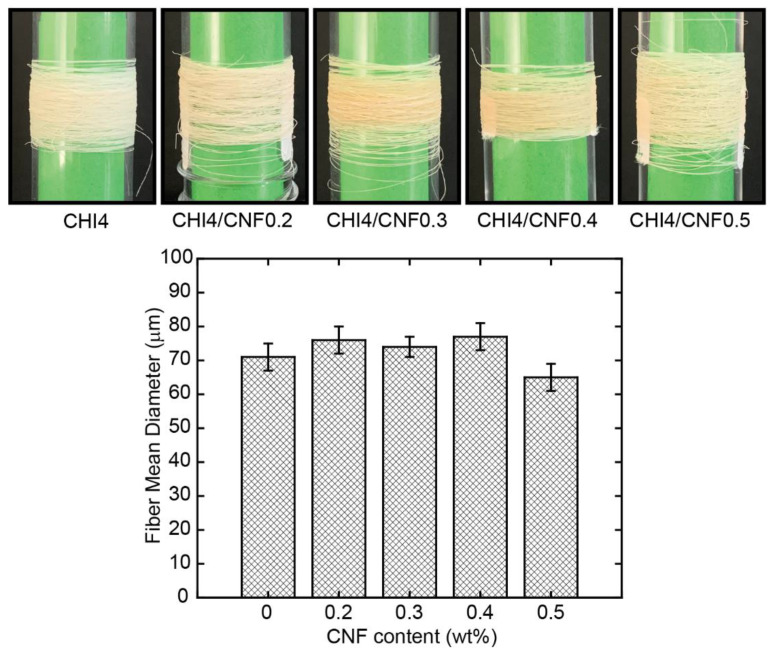
(**Top**) Photos of the obtained CHI/CNF spun fibers of varied compositions. (**Bottom**) Histogram showing the mean CHI/CNF fiber diameter obtained with the different CHI/CNF collodion formulations.

Figure 4 shows the physical appearance, as visually observed, of the spun fibers obtained by gel spinning,. Pure CHI fibers are white and have a silvery shine appearance, while CNF-filled CHI fibers present an opaque beige color with no indication of reflectance. This low coloration of the latter could be related to a slight chemical reticulation induced during fiber drying by heating, with this phenomenon enhanced in the composites. The mean diameter of the obtained CHI/CNF fibers ranged between 60 and 80 microns, with a slight increase trend observed with CNF content augmentation to 0.4 wt %. Then, for further CNF concentration, the diameter started to slightly decrease.

**Figure 5:**
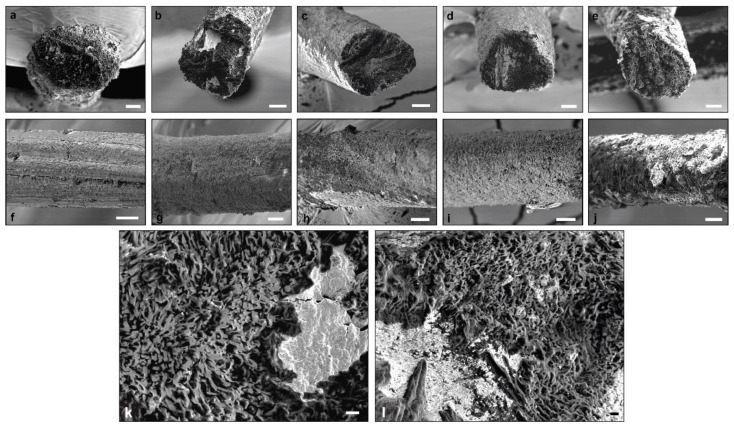
(Top) SEM micrographs of the lateral and fracture cross-section surface of the spun CHI/CNF composite fibers of: (**a**,**f**) CHI4; (**b**,**g**) CHI4/CNF0.2; (**c**,**h**) CHI4/CNF0.3; (**d**,**i**) 4CHI/CNF0.4 and (**e**,**j**) CHI4/CNF0.5. Scale bars: 20 μm. (Bottom) SEM micrographs at higher magnification of fracture cross-section surface of the spun CHI/CNF composite fibers of: (**k**) CHI4 and (**l**) CHI4/CNF0.4. Scale bars: 2 μm.

The morphology of the lateral and fracture surface of the spun CHI/CNF fibers of different compositions was closer characterized by scanning electron microscopy (SEM). Starting with pure CHI, the fiber exhibits a slightly rough and fibrillar lateral surface (Figure 5). Notin et al. [64] also described a surface nanofibril patterning in chitosan fibers spun via a pseudo-dry spinning process (in ammonia vapors) and related their formation via smaller submicron oblong objects, which were also observed in unstretched gels by small-angle light scattering and cryo-SEM. The orientation of these nanofibril-like features appeared with parallel orientation along the stretching process direction. In the here obtained CNF-filled CHI fibers, that fibrillary patterning on the lateral surface was not distinguished. Nevertheless, in the cross-section inner microstructure, a submicron fibril and porous sponge-like assembly were observed, highlighted at higher SEM magnifications (Figure 5, bottom). In the micrographs, some white regions were observed, which could be due to the presence of some remaining salts. For example, sodium acetate was produced in the neutralization step, which was not completely removed during fiber washing. The lateral surface of the CNF-filled CHI composite fibers also shows a porous microstructure, which was less homogeneous at the highest CNF content of 0.5 wt % perhaps related to a nonhomogeneous dispersion of the CNF in the initial suspension and hydrogel precursor states, when using higher CNF contents. It has been reported that fiber heating, for example, by using radiant heat, such as a radiator or heated chrome rollers, could introduce damages to the fiber surface while generating cracks as a result of the elevated thermal stress [65]. Such effects are not expected here since we used hot airflow at a temperature below the degradation temperature of both polymers (see ATG study below).

### 3.3. Fourier-Transform Infrared Spectra of the CHI/CNF Fibers

**Figure 6:**
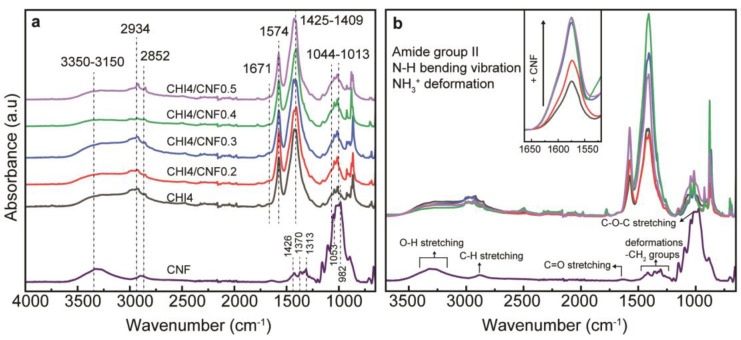
(**a**) ATR-FTIR spectra of CHI/CNF spun fibers, with the reference spectrum of the CNF alone. (**b**) Closer view to the amide II band region around 1550–1650 cm^−1^, after comparison of all FTIR spectra as in (**a**).

The chemical structure of the CHI/CNF fibers, including the interaction of CHI and CNF in the fibers, was investigated by attenuated total reflection–Fourier transform infrared spectroscopy (ATR-FTIR) as displayed in Figure 6. The spectra of the CHI/CNF fibers exhibit a broad absorption band around 3350–3150 cm^−1^, corresponding to the O–H and N–H stretching, which indicates the formation of intermolecular H-bonds in both CNFs and CHI [45]. The peaks at 2934 and 2852 cm^−1^ are attributed to the asymmetric and symmetric stretching vibrations of the C–H group, respectively [66]. The characteristic absorption of the amide I band in CHI was observed as a shoulder at 1671 cm^−1^, representing the C=O stretching [67,68]. The amide II band displays its peak at 1574 cm^−1^, which corresponds to N–H bending vibration, C–N stretching vibration and even the NH_3_^+^ symmetric deformation. The increase of the CNF content in the composite fibers resulted in an appreciable increase of the intensity of this amide band, indicating favorable interaction between the COO^−^ group of cellulose and the amine group of CHI [66,69]. In the pure CHI fibers, this amide signal should be related to the N-acetylated glucosamine units present in the CHI copolymer. For comparison, the spectra of the spun CHI fiber, a produced CHI acetate film, and the starting CHI power are shown in Figure S1 of Supplementary Material. Coming back to the FTIR spectra of Figure 6, the methylene and methyl groups are visible between 1425–1409 cm^−1^, which contribution also increased, as expected, at augmenting the CNF content. Then, the stretching of the C–O groups from primary and secondary hydroxyl group present their characteristic bands at 1044 and 1013 cm^−1^, respectively [69,70]. Finally, CHI, cellulose (e.g., CNF) and others polysaccharides show a fingerprint at the range between 1250 and 800 cm^−1^. As a comparison, Figure 6 also presents the spectra of CNF showing similarities with those of CHI (as both polymers are linear polysaccharides with similar structure), but indeed without the amine and amide absorption bands, which latter might reflect the CNF–CHI interaction. Moreover, the slight coloration observed in the spun fibers containing CNF also could be related to the amide formation crosslinking between CHI and CNF, which should contribute to the above-mentioned polymer reticulation promoted by heating.

### 3.4. Crystalline Microstructure of CHI/CNF Fibers. Wide-Angle X-ray Scattering

**Figure 7:**
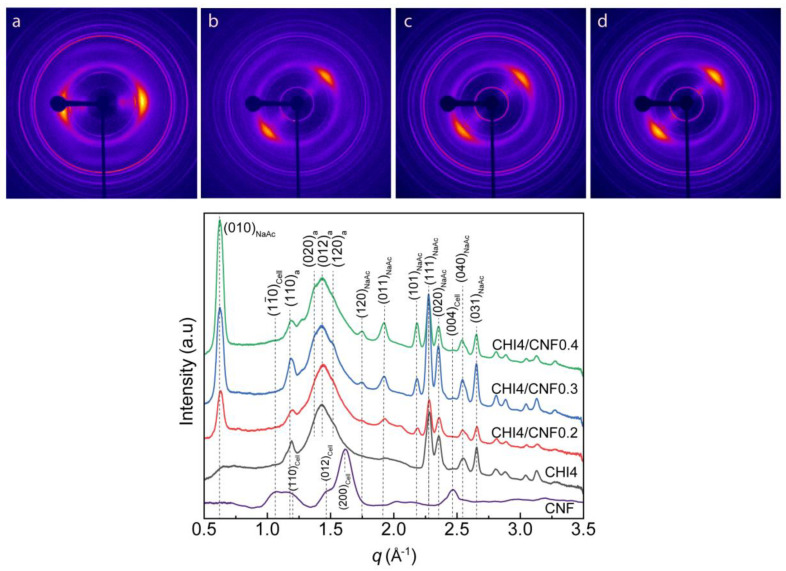
(**Top**) Two-dimensional wide-angle X-ray scattering (2D-WAXS) images of the spun CHI/CNF fibers of (**a**) CHI4, with vertical fiber axis; (**b**) CHI4/CNF0.2, (**c**) CHI4/CNF0.3 and (**d**) CHI4/CNF0.4 with fiber axis tilted by −45° from the vertical position. (**Bottom**) Radial average over the 360° azimuth of the above 2D-WAXS images of spun fibers of varied CHI/CNF compositions. In the peak indexations, the notations “a”, “Cell”, and “NaAc” refer to the corresponding reflections of chitosan anhydrous polymorph, cellulose I allomorph, and sodium acetate salt crystals, respectively.

Figure 7 shows the characterization of the crystalline microstructure by wide-angle X-ray scattering (WAXS) of the obtained CHI/CNF spun fibers. The diffraction arcs present in the 2D-WAXS diffractograms (Figure 7) show the preferential orientation of both the nanofiber reinforcement CNF, the chitosan polymer chain and produced CHI crystals in the direction of fiber stretching, demonstrating the anisotropic microstructure of the fibers.

Figure 7 also shows the X-ray scattering curves resulting from the radial average of the analyzed 2D-WAXS images. The diffraction patterns reveal signals at the scattering vector *q* around 1.2, 1.4 and 1.5 Å^−1^, attributed to crystallographic reflections (110), (020), (012) and (120) of the chitosan anhydrous allomorph (denoted by “a” in the peak indexation shown in Figure 7) [49,71,72,73]. The coagulation and here considered processing into dry fibers promote the crystallization of CHI into the anhydrous crystalline allomorph. The sequential acidic-basic-neutral treatments of the CHI-based viscous collodion should promote the hydrophobic interactions in chitosan chains, supporting their self-assembly into anhydrous crystalline allomorph, as previously proposed by Osorio et al. [49,72,73,74], when investigating the re-crystallization of chitosan chains during their acid hydrolysis at the solid-state. In the spinning process, this phenomenon should initially occur at the collodion coagulation step, continue during the stretching, washing, further stretching and finally fiber drying, yielding highly crystalline fibers with a major contribution of chitosan anhydrous allomorph. Ogawa et al. [75], when describing the new anhydrous chitosan polymorph in 1984, reported that this is energetically more stable because of additional interchain hydrogen bonding formed upon removal of loosely bound water molecules between chains along the [010] direction compared to the hydrated chitosan polymorph [75,76]. When considering the spinning collodion system incorporating cellulose nanofibers, these latter also should serve as a template for the nucleation and growth of CHI crystals as chitosan anhydrous allomorph [73,77]. Then, during the spinning process, the neutralization of the chitosan acetate (chitosan + acetic acid) hydrogel with NaOH yields sodium acetate (NaAc). In the diffractograms of Figure 7, the relatively sharp peaks at *q* ∼0.6, 1.9, 2.5 and 2.6 Å^−1^ correspond to reflections of the remaining sodium acetate salt, which might not be completely removed during fiber washing as observed in SEM micrographs (see Figure 5 displaying some mineral white spots). The intensity of these signals increases with the increase of the CNF content, which may be related to a barrier effect of the cellulose nanofibers, which slows the removal of the sodium acetate by diffusion during washing with water. An extension of the spinning setup, incorporating an elongated washing bath or more washing steps, should improve the purity of the fibers [78].

### 3.5. Thermogravimetric Analysis

**Figure 8:**
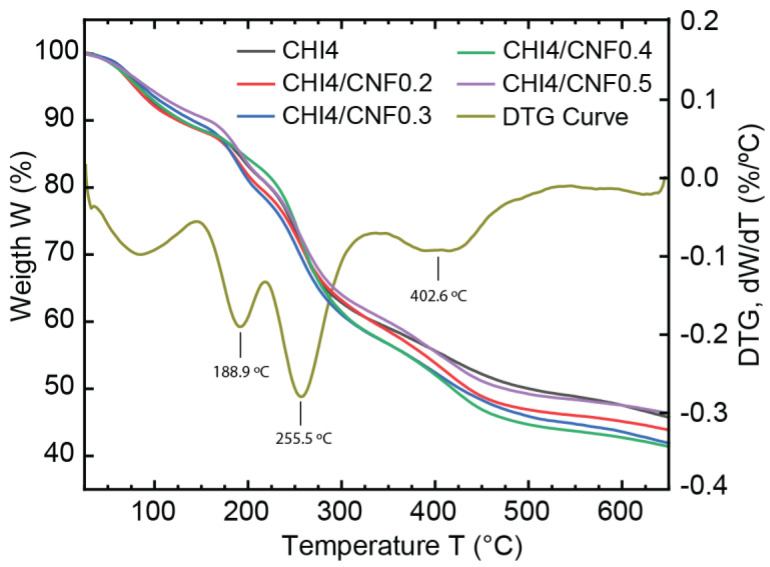
Thermogravimetric analysis (TGA) and derivative thermogravimetry (DTG, exemplified to analyze the nanocomposite fiber CHI3/CNF0.3) of the CHI/CNF spun fibers of varied compositions.

The thermal stability and decomposition patterns of pure CHI and CNF-filled CHI fibers were investigated through thermogravimetric analysis (TGA), as shown in Figure 8. After an initial weight loss of the samples around 100 °C [79], attributed to the desorption of water molecules bound to the hydrophilic polysaccharide structures, the TGA curves of almost all spun fiber compositions (from collodions CHI4, CHI4/CNF0.2, CHI4/CNF0.3, CHI4/CNF0.5) show three thermal degradation steps at around 189, 256 and 403 °C. The weight loss at 189 °C might correspond to the degradation of losing chitosan polymer chain ends or possible produced oligomers as previously reported for this temperature range [79]. Then, the main decomposition of the repeating units of polysaccharides like chitosan and cellulose is observed at around 256 °C [65,79,80]. Afterward, a minor decomposition was observed at ~400 °C, which may correspond to the degradation of remaining sodium acetate, as also suggested from SEM and WAXS analyses. It is worth noticing, for the formulation CH4/CNF0.4, the thermal stability was extended to ~256 °C, at which temperature the first degradation step was observed, in contrast to all other samples, which showed an early decomposition at ~189 °C. For cellulose nanofiber-filled polymer materials, it has been shown that the content of polysaccharide nanofibers in the composite plays a major role in the strengthening effect and thermal properties of the nanofibers [81]. The thermal stability improvement observed for this formulation CH4/CNF0.4 seems to be related to an achieved threshold of concentration of nanoreinforcement, at which the matrix/filler and filler/filler interactions seem to be optimum to positively influence the morphological and thermal properties of the CHI/CNF composite [79], This might correspond to the threshold of CNF content at which a fibril percolation effect should occur with the formation of a network of nanofibers within the polymer matrix, resulting in thermal stabilization of cellulose nanofiber-filled composites [82].

### 3.6. Dynamic Mechanical Thermal Analysis (DMTA)

**Figure 9:**
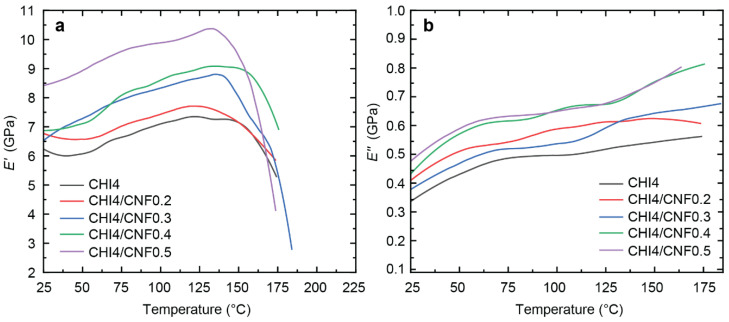
(**a**) Storage modulus (*E′*) and (**b**) loss modulus (*E″*) as a function of temperature (T) of CHI/CNF fibers.

The temperature effect on the mechanical behavior of the CHI/CNF composite fibers was investigated by DMTA. We investigated the influence of filler on possible macromolecular relaxation processes or structural transitions of the materials [83,84]. Figure 9 shows the evolution of the storage modulus *E′* and the loss or dissipation modulus *E″* with temperature for pure chitosan fiber and CNF-filled CHI composite fibers of varied CNF contents.

A similar DMTA pattern was observed for the different samples, but with an enhanced reinforcement at increasing the CNF content. The addition of CNFs, from 0.2% to 0.5% in the spinning precursor formulation, notably increases the storage modulus *E′* in the glassy region, which was extended till ~137 °C. Table 3 shows *E′* values obtained for the different CHI/CNF fiber compositions in this region, for example, at 77 °C (Figure 9), showing the increase of *E′* from 6.6 till 9.6 GPa by only adding 0.5% of CNF to the CHI formulation. The high reinforcing effect can be associated with (i) the orientation of cellulose fibers and (ii) interaction and crosslinking of chitosan at their surface, resulting in forming a hybrid CHI/CNF composite network [85]. Since Young’s modulus is sensitive to interphase interactions, it can be concluded that there is indeed good compatibility in the mixture of CHI and CNF to form nanocomposite fibers. Still, in the glassy region, a slight increase of the storage modulus with the temperature was observed, suggesting some water desorption and possibly further chemical reticulation of the polymer material due to heating above 130 °C. Afterward, at ~150 °C, an abrupt drop of the *E′* modulus was observed for all the curves that continued until their respective rupture. This modulus decay around this temperature could be related to the onset of possible relaxation of the chitosan chains. Discussion of the molecular mobility in chitosan, it is difficult to suggest the existence of a defined glass transition temperature *T_g_* as many hydrogen-bond interactions are involved in the highly crystalline linear polysaccharide matrix. However, some literature claims such a relaxation exists between 150 to 200 °C [86].

### 3.7. Micromechanical Properties

**Figure 10:**
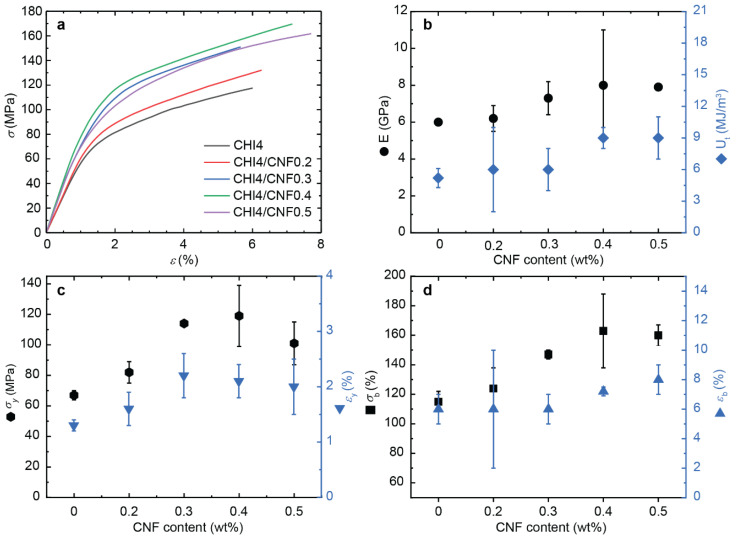
Tensile mechanical properties of spun CHI/CNF fibers obtained for different formulations. (**a**) Nominal stress–strain curves. Evolution of (**b**) Young’s modulus *E* and toughness *U_t_*, (**c**) the stress (*σ_y_*) and strain (*ε_y_*) values at the yield point, (**d**) the stress (*σ_b_*) and strain-at-break (*ε_b_*), with the increase of CNF content.

Figure 10 shows the stress–strain curves of the spun CHI/CNF fibers obtained for the different formulations, including the plot of the determined Young’s modulus *E*, the yield stress *σ_y_* and strain *ε_y_*, the stress-at-break *σ_b_* (ultimate strength), the strain-at-break *ε*_b_, and the toughness *U_t_*.

Both tensile strength and stiffness gradually increase when the CNF content in the composite fibers increases up, related to the reinforcing effect of the nanofibers. Compared to the pure CHI fibers, the addition of 0.4 wt % of CNF in the formulation led to a maximum in the values of *E*, *σ_y_* and *σ_b_*. Despite a low CNF content added to the CHI matrix, *E* and *σ_b_* already show a strong increase of 33% and 42%, respectively. The orientation and high aspect ratio of the reinforcing nanofibers (i.e., average length divided by diameter) allow for a good stress transfer from the matrix to the fibers and thereby an optimal mechanical resistance, supporting the concept of composites and their choice in advantage to single-component materials [87,88]. Doench et al. [14] evidenced that during the viscous processing of the CNF-filled CHI suspensions, balanced interactions could be established between CHI (polycation) and CNF (with surface polyanionic charge), in addition to hydrogen bonding interactions between the -OH and -NH functional groups of cellulose and chitosan. This balance allows for good filler/matrix compatibility, maintaining an homogeneous dispersion of CNFs within the CHI matrix and strong interfacial interactions between both polysaccharides, which facilitates efficient stress transfer [79,89]. Finally, *U_t_* increased with the CNF content increase (Figure 10b), as the plasticity and stress hardening processes prevail in the presence of both the CHI polymer matrix and the nanofiber filler (CNF). A possible plasticity mechanism could be nanofibers (CNF) slippage, where continuous breakage and reformation of ionic and hydrogen bond interactions between CHI and CNF could occur. Previous works showed that this dissipative process could result in a notable toughness increase [45]. In many reported studies on composite nanoreinforcement, a decrease of ductility has been observed upon addition of CNFs. The nanofibers generally increased stiffness but at the expense of the strain-at-break decrease in composite systems. In the here proposed work, the strain-at-break of the spun CNF-filled CHI fibers were similar or even higher than that of the pure CHI fibers (Figure 9). Similar synergistic behavior was observed by Dogan et al. [90] and Tang et al. [91] In the present work, the spun fibers with the highest CNF content (0.4% and 0.5%) showed a significantly enhanced strain-at-break, which reflects the very good interface between the CNFs and the chitosan matrix [92,93].

To summarize, the matrix/filler and filler/filler interactions play major roles in the strengthening effect of cellulose nanofibers in chitosan fiber materials [81]. It has been shown that for low levels of polysaccharide nanofibers in composite, the matrix/filler interaction plays the main role; while for higher nanofiber contents, the excellent mechanical properties have been explained by strong fibril/fibril interactions and also by a percolation effect [82]. These interactions have been proposed to mainly take place through hydrogen bonds that are established during the solvent evaporation to yield composites, which allows forming a network of nanofibers within the polymer matrix [82]. According to the percolation model, the threshold of nanofiber content needed to achieve their percolation (percolation threshold) strongly depends on the dispersed phase aspect ratio (L/d) and its spatial orientation. The nanofibrillated cellulose filler, as used in this work, presents an extremely high aspect ratio, which supports the fact that a very low fraction of CNFs is needed to achieve chitosan-based fibers of high performance. Here, we show that a CNF content of around 0.4 wt %, dispersed in a 4 wt % viscous chitosan formulation, is optimum to spin chitosan-based fiber composites of excellent mechanical properties, showing Young’s modulus as high as 8 GPa, ultimate strength of 163 MPa, and toughness of 9 MJ.m^−3^.

## 4. Conclusions

Functional chitosan-based fibers were achieved by incorporating small amounts of nanofibrillated cellulose (CNF) into viscous chitosan (CHI) collodion solution in a gel spinning process to yield anisotropic nanofiber-reinforced biocomposite fibers of excellent mechanical properties. The addition of the CNFs in the CHI matrix plays an important role in the spun composite fiber morphology, thermal and mechanical properties. The CNFs significantly improved the stiffness, tensile strength, ductility and finally the toughness of CHI-based fibers, thanks to the outstanding mechanical properties and high aspect ratio of the cellulose nanofiber reinforcement as well as its good compatibility with chitosan, leading to strong interfacial adhesion between both components with involved both physical and covalent interactions. Moreover, the CNFs acted as a nucleating agent for the crystallization of the CHI polymer chains constituting the matrix, as demonstrated by 2D X-ray diffraction analysis. This phenomenon, together with the orientation of both the cellulose nanofibers and the chitosan polymer chains along the spun fiber direction in the wet/gel spinning process, should have further contributed to the reinforcement of the composite. The composite fibers of these natural polymers chitosan and cellulose as achieved here are promising as renewable, bioactive, biocompatible and mechanical performant materials for applications in biomedicine and (bio)technology. Particularly, the enhanced mechanical behavior of the CNF-filled CHI nanocomposite fibers, together with their excellent biocompatibility and tuned biodegradability, set them as outstanding candidates for biomedical applications, such as wound dressings, suture threads, knitted fabrics, tissue engineering, etc. We envisage applying these fibers in the engineering of mechanically demanding tissues like the annulus fibrosus region of the intervertebral disc, which progresses will be reported in further publications.

## 5. Patents

Osorio-Madrazo, A.; David, L.; Montembault, A.; Viguier, E.; Cachon, T. Hydrogel Composites Comprising Chitosan and Cellulose Nanofibers. International Patent Application No. WO 2019/175279 A1, 19 September 2019, US Patent Application 16/980383, 18 February 2021.

## Figures and Tables

**Table 1:** Linear speed of the roller bobbins integrated into the spinning motors.

Motor Nr.	Bobbin Linear Speed (mm/s)
1	13.1
2	15.3
3	18.1
4	22.1

**Table 2:** Flow parameters determined from the fitting of the viscosity vs. shear rate curves of different CHI/CNF collodion formulations (Figure 3), by using the Cross model (Equation (1)) for naked CHI solution and a double Cross model (Equation (2)) for CHI/CNF formulations.

**Formulation**	$\eta_{0,CHI}$ **(Pa.s)**	$\tau_{CHI}$ **(s)**	$p_{CHI}$
CHI4	1200	1.4	0.89
**CHI4 with the addition of different CNF contents:**	$\eta_{0,CNF}$ **(Pa.s)**	$\tau_{CNF}$ **(s)**	$p_{CNF}$
CHI4/CNF0.2	-	-	-
CHI4/CNF0.3	273	3.4	0.93
CHI4/CNF0.4	336	2.3	0.93
CHI4/CNF0.5	607	2.1	1.48

**Table 3:** Storage modulus *E′* obtained at 77 °C in DMTA measurements (Figure 7) of spun CHI/CNF fibers of varied compositions.

Fiber Sample	*E′* (GPa) at 77 °C
CHI4	6.6
CHI4/CNF0.2	7.0
CHI4/CNF0.3	7.9
CHI4/CNF0.4	8.1
CHI4/CNF0.5	9.6

## Supplementary Materials

The following are available online at https://www.mdpi.com/article/10.3390/polym13101563/s1, Figure S1: FTIR spectra of starting chitosan powder, and after its processing into chitosan fiber, and into chitosan acetate film.
