# Supplementary material for: Functional Bionanocomposite Fibers of Chitosan Filled with Cellulose Nanofibers Obtained by Gel Spinning
Source: Polymers (Basel). 2021 May 13;13(10):1563. doi: 10.3390/polym13101563 (PMC8152965; doi:10.3390/polym13101563)
Supplement: Supplementary file 1 [file polymers-13-01563-s001.zip › polymers-1180663-supplementary.pdf]

Supplementary Material

Functional Bionanocomposite Fibers of Chitosan Filled with Cellulose Nanofibers Obtained by Gel Spinning

Sofia Marquez-Bravo [1,2,3], Ingo Doench [1,2,3], Pamela Molina [1,2,3], Flor Estefany Bentley [1,2,3], Arnaud Kamdem Tamo [1,2,3], Renaud Passieux [4], Francisco Lossada [5], Laurent David [4] and Anayancy Osorio-Madrazo [1,2,3,*]

[1] Institute of Microsystems Engineering IMTEK, University of Freiburg, 79110 Freiburg, Germany; sofia.marquez@imtek.uni-freiburg.de (S.M.-B.); ingo.doench@imtek.uni-freiburg.de (I.D.); molina@tf.uni-freiburg.de (P.M.); estefany.bentley@imtek.uni-freiburg.de (F.E.B.); arnaud.kamdem@imtek.uni-freiburg.de (A.K.T.)

[2] Freiburg Materials Research Center FMF, University of Freiburg, 79104 Freiburg, Germany

[3] Freiburg Center for Interactive Materials and Bioinspired Technologies FIT, University of Freiburg, 79110 Freiburg, Germany

[4] Laboratoire Ingénierie des Matériaux Polymères IMP, CNRS UMR 5223, University Claude Bernard Lyon 1, University of Lyon, 69622 Villeurbanne Cedex, France; renaud.passieux@etu.univ-lyon1.fr (R.P.); laurent.david@univ-lyon1.fr (L.D.)

[5] Department of Chemistry, University of Mainz, 55128 Mainz, Germany; francisco.lossada@uni-mainz.de

* Correspondence: anayancy.osorio@imtek.uni-freiburg.de; Tel.: +49-761-203-67363

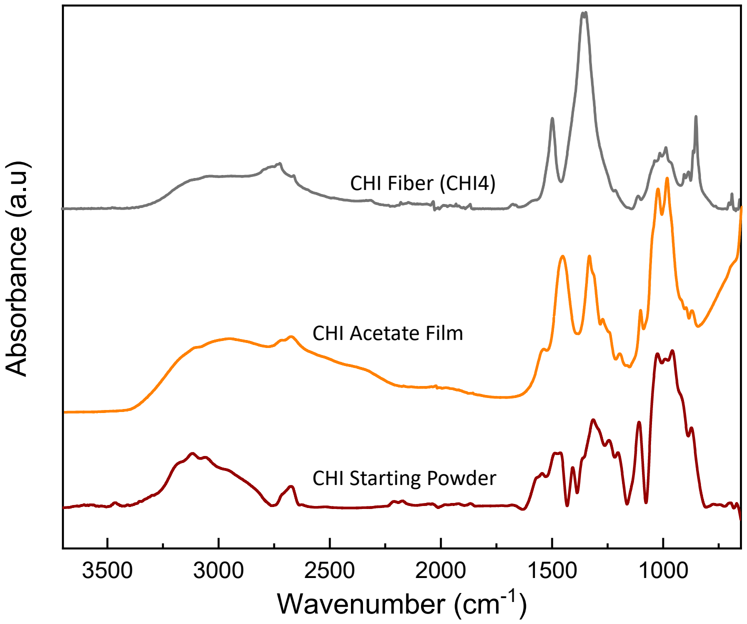

Figure S1. FTIR spectra of starting chitosan powder, and after its processing into chitosan fiber (formulation CHI4% in the manuscript) and into chitosan acetate film materials.
